# RGS10 deficiency ameliorates the severity of disease in experimental autoimmune encephalomyelitis

**DOI:** 10.1186/s12974-016-0491-0

**Published:** 2016-02-01

**Authors:** Jae-Kyung Lee, George T. Kannarkat, Jaegwon Chung, Hyun Joon Lee, Kareem L. Graham, Malú G. Tansey

**Affiliations:** Department of Physiology and Pharmacology, College of Veterinary Medicine, University of Georgia, 501 D.W. Brooks Dr, Athens, GA 30602 USA; Department of Physiology, Emory University School of Medicine, Atlanta, GA USA

**Keywords:** Regulator of G-protein signaling (RGS10), Neuroinflammation, Monocyte, T cell, Adoptive transfer, Experimental autoimmune encephalomyelitis (EAE), Demyelination

## Abstract

**Background:**

Regulator of G-protein signaling (RGS) family proteins, which are GTPase accelerating proteins (GAPs) that negatively regulate G-protein-coupled receptors (GPCRs), are known to be important modulators of immune cell activation and function. Various single-nucleotide polymorphisms in RGS proteins highly correlate with increased risk for multiple sclerosis (MS), an autoimmune, neurodegenerative disorder. An in-depth search of the gene expression omnibus profile database revealed higher levels of RGS10 and RGS1 transcripts in peripheral blood mononuclear cells (PBMCs) in MS patients, suggesting potential functional roles for RGS proteins in MS etiology and/or progression.

**Methods:**

To define potential roles for RGS10 in regulating autoimmune responses, we evaluated RGS10-null and wild-type (WT) mice for susceptibility to experimental autoimmune encephalomyelitis (EAE), a widely studied model of MS. Leukocyte distribution and functional responses were assessed using biochemical, immunohistological, and flow cytometry approaches.

**Results:**

RGS10-null mice displayed significantly milder clinical symptoms of EAE with reduced disease incidence and severity, as well as delayed onset. We observed fewer CD3+ T lymphocytes and CD11b+ myeloid cells in the central nervous system (CNS) tissues of RGS10-null mice with myelin oligodendrocyte protein (MOG)_35–55_-induced EAE. Lymph node cells and splenocytes of immunized RGS10-null mice demonstrated decreased proliferative and cytokine responses in response to in vitro MOG memory recall challenge. In adoptive recipients, transferred myelin-reactive RGS10-null Th1 cells (but not Th17 cells) induced EAE that was less severe than their WT counterparts.

**Conclusions:**

These data demonstrate a critical role for RGS10 in mediating autoimmune disease through regulation of T lymphocyte function. This is the first study ever conducted to elucidate the function of RGS10 in effector lymphocytes in the context of EAE. The identification of RGS10 as an important regulator of inflammation might open possibilities for the development of more specific therapies for MS.

## Background

Multiple sclerosis (MS) is a chronic neurodegenerative, autoimmune disease of the central nervous system (CNS) characterized by demyelination. Over 250,000–300,000 individuals in the United States currently suffer from MS, and 200 new cases are diagnosed every week. Although it has been 170 years since its first description, its etiology remains unknown. Genetic predisposition, environmental factors, and autoimmune inflammatory mechanisms play an important role in the pathogenesis of MS [1, 2].

G-protein-coupled receptor (GPCR) signaling influences various aspects of MS pathogenesis, including antigen presentation, cytokine/chemokine production, and T cell differentiation, proliferation, and infiltration (see review [3]). GPCRs signal through heterotrimeric G proteins that consist of an α subunit and a βγ heterodimer [4]. Regulator of G-protein signaling (RGS) proteins contain an evolutionarily conserved RGS domain that interacts with a Gα_i_, Gα_q/11_, or Gα_12/13_ subunit with variable selectivity, which accelerates the GTPase activating function of Gα subunits [5–7]. RGS proteins differ widely in their size and contain a variety of structural domains in addition to the RGS domain and motifs that regulate their activity and determine regulatory binding partners [6, 8] (reviewed in [9–12]). In addition to its primary function as a negative regulator of G protein signaling, it is now appreciated that the non-RGS regions of RGS proteins can sustain non-canonical functions distinct from the inactivation of Gα subunits or even from G protein signaling entirely (reviewed in [10, 12, 13]). Larminie et al. reported that there are tissue-specific patterns of RGS proteins in human peripheral tissues and brain [14]. The RGS protein expression profile in human lymphocytes [14] is quite similar to that in rodent lymphocytes [15], although RGS protein profiles in various subsets of immune cells still need to be explored.

Recently, multiple lines of genetic evidence have highlighted roles for RGS family proteins in mediating autoimmune disease: (1) The International Multiple Sclerosis Genetics Consortium (IMSGC) identified RGS1 as a novel MS susceptibility locus [16]; (2) SNPs of RGS1, RGS7, RGS9, and RGS14 are highly correlated with a diagnosis of MS, Crohn’s disease (CD), and ulcerative colitis (UC) [16–20]; (3) The mRNA levels of RGS10 are higher in the peripheral blood mononuclear cells (PBMC) from patients with MS, CD, and UC compared to those in unaffected individuals [21]. The transcript level of RGS1 is higher in MS patients as reported in the gene expression omnibus profile database [22]. However, the mechanisms via which RGS proteins modulate the onset or progression of autoimmune diseases and/or whether RGS proteins influence lymphocyte function or migration in preclinical models of MS has never been explored. Specifically, RGS10 is one of the smallest RGS family proteins and is highly expressed in the brain, thymus, and lymph nodes [6, 23–25]. We have previously shown that RGS10 is a negative regulator of microglial and macrophage activation [26–28]. In this study, we investigated the role of RGS10 in T cells in the mouse experimental autoimmune encephalomyelitis (EAE) model of MS.

## Methods

### Mice

Generation of RGS10-null mice (C57BL/6) has been described previously [27]. The 2D2 TCR mice C57BL/6J mice were purchased from The Jackson Laboratory (Bar Harbor, ME). Eight to 12-week-old male mice were used for experiments, except where indicated. Experimental procedures involving use of animal tissue were performed in accordance with the NIH Guidelines for Animal Care and Use and approved by the Institutional Animal Care and Use Committee at Emory University School of Medicine in Atlanta, GA. Unless noted, mice were euthanized by intraperitoneal Euthasol injection.

### EAE induction

RGS10-null mice and wild-type (WT) littermates (C57BL/6 background) were actively immunized with myelin oligodendrocyte protein (MOG)_35–55_ as described to initiate EAE [29, 30]. Briefly, mice were injected subcutaneously (s.c.) with 100 μg of MOG_35–55_ peptide emulsified in complete Freund’s adjuvant (CFA) supplemented with 250 μg of heat-inactivated *Mycobacterium tuberculosis* H37 RA. In addition, mice received an intraperitoneal (i.p.) pertussis toxin injection (250 ng) at the time of sensitization and 48 h later. Mice were monitored daily for clinical signs, and disease was evaluated as described [31]. For induction of EAE by adoptive transfer, mice were injected with MOG_35–55_/CFA and pertussis toxin as described above. Splenocytes and draining lymph node cells were harvested on day 9 after immunization and expanded in vitro with 10 μg/ml of MOG_35–55_ and recombinant mouse (rm) IL-12 (10 ng/ml, R&D Systems) to induce Th1 cells or rmIL-23 (10 ng/ml, R&D Systems) to induce Th17 cells for additional 72 h. Cells were then harvested, washed once with saline, counted and injected i.p. into 5- to 6-week-old WT naïve recipient mice (10 million cells per mouse) i.p. Mice were followed clinically up to at least day 30 post-transfer.

### Tissue processing and LFB staining

At the time of sacrifice, the spinal cords were removed and fixed in 4 % paraformaldehyde for 24 h and then embedded in paraffin. Sections were cut at 20 μm on a microtome and stained by Luxol fast blue (LFB) to reveal demyelinated areas. For LFB staining, the sections were fixed in 4 % PFA for 10 min, followed by washing in 1× PBS for 5 min. The sections were cleaned by xylene for 10 min and then was hydrated in 100 % EtOH for 5 min and 95 % EtOH for 5 min. The sections were stained in Luxol fast blue solution for 1 h and 45 min, followed by a rise of 95 % EtOH for 5 min and Milli-Q water for 3 min. The sections were differentiated for 10 s in lithium carbonate solution, then 10 s in 70 % EtOH, 10 s in milli-H_2_O, and 5 s in lithium carbonate again, and 5 s in 70 % EtOH. Images of RGS10 EAE sections were captured under ×20 objective lens on a Nikon 90i microscope using thresholding analysis on image J software by an investigator blinded to treatment history.

### Mononuclear cell isolation and flow cytometry

Mononuclear cells from the spinal cord were isolated by mechanical and enzymatic dissociation methods followed by Percoll gradient (70/30 %) centrifugation [32]. T cells (CD45+CD3+), neutrophils (CD11b+Ly6G+), B cells (CD19+), myeloid cells (Ly6G-CD11b+), Th1 (CD4+T-bet+), or Th17 (CD4+RORγt+) were analyzed by flow cytometry. Flow cytometry data were acquired using an LSRII instrument and analyzed using FlowJo Software.

### T cell recall proliferation and cytokine secretion

Spleen and lymph nodes were collected from RGS10-null or WT mice at day 10 post-MOG_35–55_/CFA immunization. Single-cell suspensions were prepared by mechanical disruption in RPMI-1640 medium supplemented with 10 % FBS, 100 IU/ml penicillin, 100 μg/ml streptomycin, 1× non-essential amino acids, 1 μM sodium pyruvate, 50 mM 2-ME, and 2 mM L-glutamine; 2 × 10^5^ cells per well in a 96-well plate were activated by different concentrations of MOG_35–55_ or plate-bound anti-CD3 (5 μg/ml, 145-2C11, eBioscience) plus soluble anti-CD28 (5 μg/ml, 37.51, eBioscience) for 72 h and proliferation was assessed via MTS assay (Promega). Supernatants were collected after 72 h of culture, and cytokine levels were measured by mouse multiplexed Meso Scale Discovery ELISAs (Meso Scale Discovery) [27].

### DC and CD4+ T cell isolation and in vitro antigen presentation assay

Dendritic cells (DCs) were isolated from the spleens and lymph nodes of RGS10-null or WT mice. Tissues were incubated with CD90.2 beads to deplete T cells followed by positive selection using CD11c beads (Miltenyl Biotech). CD4+ T cells were isolated from the spleens of 6-week-old 2D2 TCR mice using the CD4+ T cell isolation kit II (Miltenyl Biotech); 2 × 10^4^ DCs were incubated with 1 × 10^5^ CD4+ T cells in the presence of different concentrations of MOG_35–55_ for 72 h, and T cell proliferation was assessed via MTS assay.

### Chemotaxis assay

A BD transwell system with a pore size of 5 μm (Corning, Lowell, MA, USA) was used for the migration assay. In the bottom compartment, CXCL-12 (10 nM, R&D System) was added in chemotaxis buffer (0.5 % BSA)/RPMI-1640. As a chemokinesis control, we included CXCL-12 (10 nM) in both the bottom and top compartments. CD4^+^ T cells (1 × 10^5^) were seeded in the top compartment, and after a 2-h incubation (37 °C, 5 % CO_2_), the inserts were removed, and the cells that had migrated through the filter to the lower chamber were counted by flow cytometry. Polystyrene beads (15.0 μm diameter, Polysciences, Warrington, PA, USA) were added to each well to allow the cell count to be normalized. A ratio was generated and percent input migration was calculated.

### In vitro T cell differentiation

Naïve T cells (CD4+CD25−) from 6–8-week-old RGS10-null or WT male mice were isolated from the spleen using Miltenyi beads. T cells (2 × 10^5^ cells/well) were incubated in flat-bottomed 96-well plates at 37 °C for 3 days with plate-bound anti-CD3 (1 μg/ml) plus soluble anti-CD28 (1 μg/ml) in the presence of (i) anti-IL-4 neutralizing antibody (10 μg/ml, 11B11, eBioscience) and rm IL-12 (10 ng/ml) for Th1 differentiation or (ii) anti-IL-4 (10 μg/ml) and anti-IFN-γ neutralizing antibodies (10 μg/ml, R4-6A2, BD Bioscience), rmIL-6 (20 ng/ml, R&D System), and human TGF-β1 (3 ng/ml, R&D System) for Th17 differentiation. Phorbol myristate acetate (PMA) (50 ng/ml) and ionomycin (1 μg/ml) plus protein transport inhibitor (eBioscience) were added for the last 5 h of the culture period. The percentages of IFNγ+ and IL-17A+ CD4+ T cells were analyzed by flow cytometry.

### Statistical analysis and power

Power analyses, outlined in the 3rd Edition of Design and Analysis, by Geoffrey Keppel, were used to estimate group sizes. The number of animals was based on our published work [33], and our studies and estimates per group for physiologic outcome measures yielded power values >0.80 with *α* = 0.05. Animal EAE scoring data were analyzed using the non-parametric Mann-Whitney *t* test. Comparison of quantitative data between two groups was assessed using the Student’s *t* test. Comparison between two groups for in vitro experiments was analyzed by two-way ANOVA followed by the Bonferroni post hoc test for *p* values. *p* < 0.05 was considered statistically significant. Specific statistical tests used for each experiment are indicated in the figure legend.

## Results

### RGS10-null mice displayed milder clinical and histological EAE

**Fig. 1:**
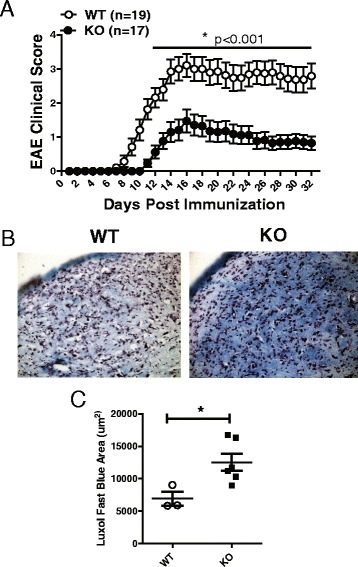
RGS10-null mice displayed attenuated EAE. EAE was induced by active immunization and mice were monitored daily for clinical disease. **a** Mean clinical scores (±SEM) of male wild type (WT, *n* = 19) and RGS10-null mice (*n* = 17). *p < 0.001, Mann-Whitney *U* test. **b** Photomicrograph and **c** quantification of CNS demyelination (Luxol fast blue staining) in WT and RGS10-null EAE mice at day 32 post-immunization. *p < 0.05, Student’s *t* test

We hypothesized that RGS10 modulates CNS autoimmunity by regulating T lymphocyte infiltration and/or effector functions. To test this possibility, we induced EAE in WT and RGS10-null mice by active immunization with MOG_35–55_. We found that RGS10-null mice induced to develop EAE by active immunization exhibited less severe clinical signs in the acute phase of disease (approximately day 9 through day 15 post-immunization (p.i.). Clinical symptoms remained mild through day 35 p.i. (Fig. 1).

Consistent with the clinical findings, LFB staining analysis and blinded measures of myelination area revealed that RGS-null mice displayed less demyelination at day 32 p.i.) (Fig. 1b, c). LFB staining in naïve CNS tissues and at day 12 p.i. showed no differences between genotypes (data not shown). In summary, RGS10-null mice had significantly lower EAE incidence and delayed onset of disease, as well as lower mean maximum clinical scores (Table 1). Potential causes for the phenotype of RGS10-null mice in EAE include, but are not limited to (1) functional defects of antigen presenting cells (APCs), such as DCs, (2) a defect in T cell proliferation or effector functions such as or cytokine secretion, (3) a defect in the expansion of autoreactive T cells, and/or (4) a defect in infiltration of effector T cells into the CNS.

**Table 1:** Clinical EAE in actively immunized WT and RGS10-null mice

	Incidence of clinical EAE (%)	Mean day of onset (SEM)	Mean maximum score (SEM)
WT	18/19 (95 %)	9.7 (0.4)	3.20 (0.3)
KO	10/17 (59 %)*	11.7 (0.3)*	1.85 (0.4)**

Data are pooled from two independent experiments

*p < 0.05, incidence of EAE and mean day of onset, determined by Fisher's exact test and Student’s *t* test respectively

**p < 0.01, mean maximum scores determined by Mann-Whitney *U* test

### RGS10-null dendritic cells efficiently stimulate CD4+ T cell activation

**Fig. 2:**
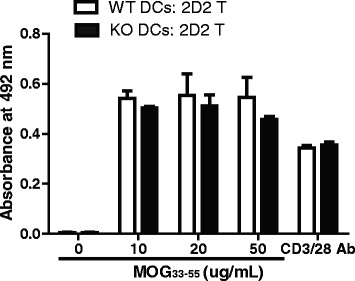
RGS10-null dendritic cells (DCs) displayed intact antigen presentation capacity. DCs derived from WT and RGS10-null mice were incubated with CD4+ T cells from 2D2 TCR mice for 72 h in the presence of the indicated concentrations of MOG_35–55._ As a positive control, CD4+ T cells from 2D2 TCR mice were stimulated in vitro with anti-CD3/CD28 (5 μg/ml) for 72 h. T cell proliferation was measured by MTS incorporation assay (*n* = 3 mice per group)

We asked if the milder EAE in RGS10-null mice was due to impaired APC function. To test this possibility, we evaluated the ability of RGS10-null or WT CD11c+ DCs to activate MOG_35–55_-specific 2D2 CD4+ T cells. We found that RGS10-null DCs stimulated proliferation of MOG_35–55_-specific 2D2 CD4+ T cells as well as their WT counterparts (Fig. 2). This suggests that peripheral RGS10-null DCs are competent in their ability to activate autoreactive CD4+ T cells.

### RGS10-null CD4+ T cells have normal proliferative and cytokine response to mitogen stimulation

**Fig. 3:**
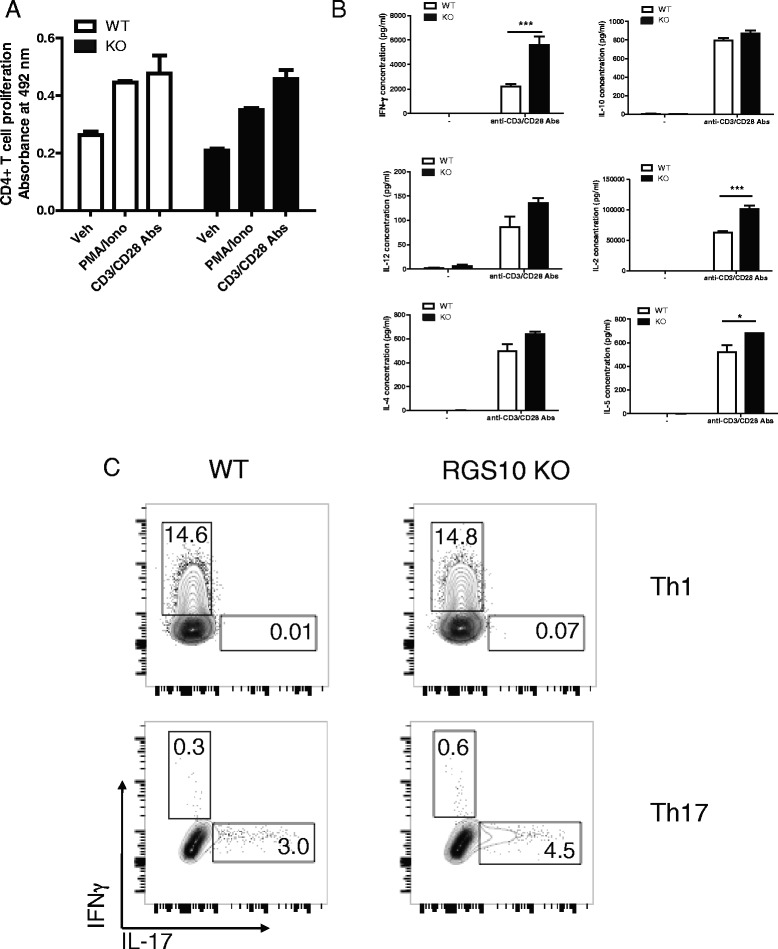
RGS10-null naïve CD4+ T cells displayed intact mitogen-mediated activation and polarization into Th1 or Th17. **a** CD4+ T cells were isolated from spleens of WT and RGS10-null mice. Cells were treated with PMA (20 ng/ml)/ionomycin (1 μM) or anti-CD3/CD28 (5 μg/ml) in vitro for 72 h. Proliferation was measured by MTS incorporation assay and (**b**) cytokine production was measured by multiplexed immunoassays (MSD). *, *p* < 0.05, ***, *p* < 0.001, two-way ANOVA. **c** Splenic naive T cells (CD4+ CD25−) were isolated from WT and RGS10-null mice. Cells were differentiated in vitro under Th1 (*top panels*) or Th17 (*bottom panels*) polarization conditions as described in the “Methods” section. PMA/ionomycin and protein transport inhibitors were added for the last 5 h of the culture period. Cells were then stained with mAbs to surface markers and intracellular cytokines and analyzed by flow cytometry. CD4+ T lymphocytes were evaluated for IFNγ and IL-17A expression. Representative FACS plots indicate the percentage of CD4+ T cells that stained positive for IFNγ or IL-17A. Two independent experiments with similar results were performed (*n* = 2–3 mice in each experiment)

We have previously shown that young (3–7-month old) RGS10-null mice do not display any abnormalities in immune cell distribution within the CNS and peripheral lymphoid tissues [34]. We found no differences between naive WT and RGS10-null mice with respect to absolute numbers of CD4+ and CD8+ T cells, B cells, and monocytes within the spleens, blood, and brain [34]. To determine whether CD4+ T cells from RGS10-null mice have defects in their ability to respond to mitogenic stimuli, we stimulated CD4+ cells from RGS10-null vs. WT mice with either (i) anti-CD3/CD28 antibodies or (ii) a combination of PMA and ionomycin (Fig. 3). In response to these mitogens, CD4+ T cells from RGS10-null mice proliferated to the same extent as cells from WT animals. In fact, CD4+ T cells from RGS10-null animals secreted higher levels of IFN-γ, IL-2, and IL-5 compared to WT cells. Thus, RGS10-null CD4+ T cells do not exhibit defects in proliferation or cytokine secretion when stimulated non-specifically through the T cell receptor (anti-CD3/anti-CD28) or when the T cell receptor is bypassed via activation with phorbol ester and calcium ionophore (PMA/ionomycin). Collectively, our data suggest that RGS10-null T cells do not have inherent, global defects in TCR signaling and that RGS10-null DCs are competent in their ability to present antigen to T cells.

Next, we asked whether RGS10-null T cells were capable of differentiating into Th1 or Th17 effector cells. For this, naive T cells from RGS10-null and WT mice were isolated and then differentiated into Th1 or Th17 cells under in vitro polarization conditions [35]. We found similar proportions of IFNγ+ and IL-17+ cells among RGS10-null vs. WT CD4+T cells that were differentiated under Th1 or Th17 conditions, respectively. Thus, CD4+ T cell-expressed RGS10 is dispensable for Th1 and Th17 differentiation in vitro (Fig. 3c).

### RGS10-null lymphocytes proliferate and produce cytokines at significantly lower levels than WT LN cells in MOG-recall assay

**Fig. 4:**
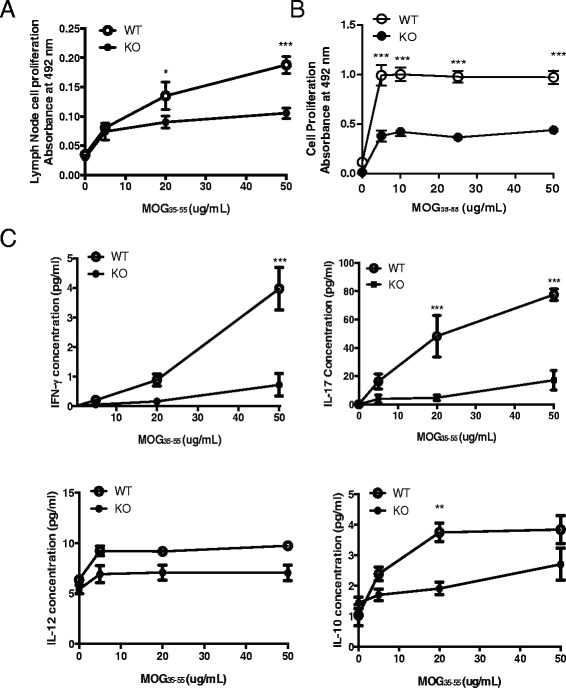
RGS10-null lymphocytes displayed attenuated recall response to MOG_35–55_. WT and RGS10-null mice were immunized with MOG_35–55_ as described in the “Methods” section. Ten days later, (**a**) lymph node cells or (**b**) splenocytes were isolated and re-stimulated ex vivo with the indicated concentrations of MOG_35–55_ peptide for 72 h. Proliferation was assessed via MTS incorporation assay. **c** After 72 h, cytokine secretion by WT and RGS10-null MOG_35–55_-re-stimulated lymph node cells was determined by MSD assay. **p < 0.01, ***p < 0.001, two-way ANOVA with Bonferroni post hoc test (*n* = 3–4 mice per group)

To further characterize T cell proliferation and effector functions in RGS10-null animals, we examined autoantigen-specific recall responses. RGS10-null or WT mice were immunized with MOG_35–55_. Ten days later, draining (inguinal) lymph node cells and splenocytes were isolated and re-stimulated with MOG_35–55_ for 3 days. Upon re-stimulation with MOG_35–55_ in vitro, draining lymph node cells and splenocytes from RGS10-null mice proliferated less and produced cytokines at levels significantly lower than their WT counterparts (Fig. 4a, b). Lymph node cells and splenocytes from RGS10-null mice produced cytokines including IFN-γ, IL-17, and IL-10 at levels significantly lower than WT cells upon re-stimulation with MOG_35–55_ in vitro (Fig. 4c). These data indicate that RGS10-null mice might have an impairment in the generation or maintenance of autoreactive T cell populations after initial activation, which may contribute to the attenuated EAE phenotype in RGS10-null mice.

### RGS10-null mice displayed attenuated neuroinflammation

**Fig. 5:**
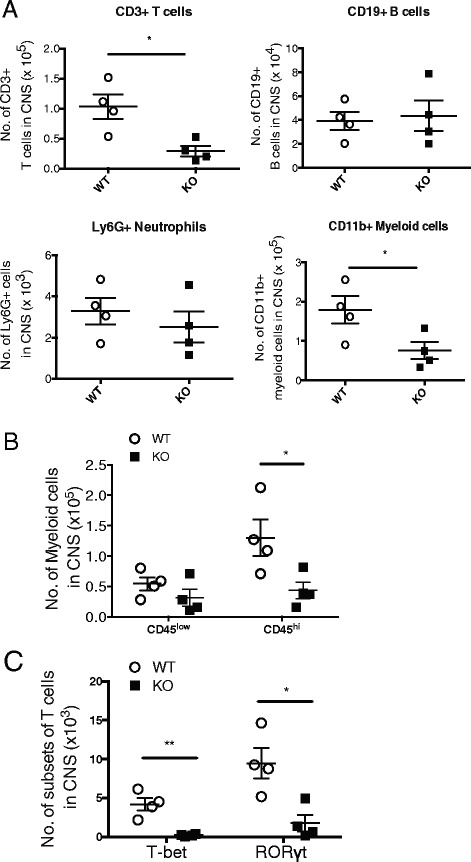
RGS10-null mice with symptomatic EAE have fewer leukocytes in the CNS. Mononuclear cells from the spinal cord were isolated from MOG_35–55_-immunized WT (*n* = 3–4) and RGS10-null (*n* = 4) mice at day 15 p.i. as described in the “Methods” section. Cells were then stained with mAbs to surface markers and/or intracellular cytokines and analyzed by flow cytometry. **a** Plot shows the CD3+ T cells, CD19+ B cells, Ly6G+ neutrophils, and CD11b+ myeloid cells among CD45+ gated cells in the spinal cord. *, *p* < 0.05, Student's *t* test. **b** Plot shows the CD45^low^ (microglia) and CD45^high^ (macrophages or activated microglia) cells among Ly6G-CD11b+ MHCII+ gated cells. *, *p* < 0.05, two-way ANOVA. **c** Plot shows the T-bet+ (Th1) and RORγt+ (Th17) cells among CD3+ CD4+ gated cells. *, *p* < 0.05, **, *p* < 0.01, two-way ANOVA

Next, we examined cellular infiltration within the CNS and the peripheral lymphoid tissues of RGS10-null and WT mice. We explored the possibility that milder EAE in RGS10-null mice could be due to reduced leukocytic infiltration into the CNS. We found no significant difference in the percentages and numbers of Th1 or Th17 cells in draining the lymph node and spleen at day 15 p.i. between genotypes, as determined by flow cytometry (data not shown). However, there were significantly fewer infiltrating leukocytes (CD45+), specifically, CD3+ T cells and CD11b+ myeloid cells in CNS tissues of RGS10-null mice. Conversely, the spinal cords from RGS10-null and WT mice contained comparable numbers of B cells and neutrophils (Fig. 5a). We also found that the fraction of CD45^high^ myeloid cells among CD11b+ gated cells in RGS10-null EAE mice is reduced relative to that in WT EAE mice, which suggests that there are relatively fewer activated microglia or infiltrating macrophages in the CNS of RGS10-null EAE mice compared to those of WT EAE mice (Fig. 5b). We also examined the composition of T lymphocytes within the CNS. Consistent with our observations of fewer CD3+ CD4+ T cells in RGS10-null CNS tissues (Fig. 5a), we found that RGS10-null CNS tissues contained significantly fewer Th1 (T-bet+) cells and Th17 (RORγt+) cells than their WT counterparts (Fig. 5c). Notably, the relative proportion of T-bet+ and RORγt+ cells within the CD4+ T lymphocyte population did not significantly differ between WT and RGS10-null mice, suggesting that RGS10 does not preferentially impact Th1 vs. Th17 differentiation within the CNS (data not shown). Collectively, our results demonstrate that there is significantly reduced inflammation in the CNS of RGS10-null mice induced to develop EAE, which is consistent with the reduced demyelination observed in RGS10-null mice with chronic EAE (day 32 p.i.) (Fig. 1b, c).

### RGS10-null CD4+ T cells displayed attenuated chemotaxis

**Fig. 6:**
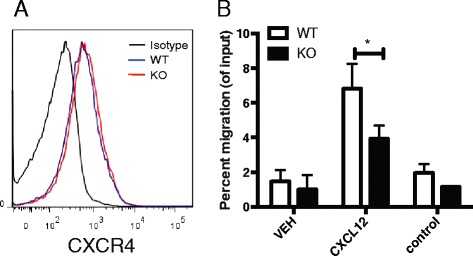
RGS10-null CD4+ T cells displayed attenuated migration to CXCL12, a ligand for CXCR4. CD4+ T cell migration to CXCL12 was tested in an in vitro transwell chemotaxis assay. **a** Histogram shows the levels of chemokine receptor CXCR4 staining on CD3+ CD19− CD4+ T lymphocytes from WT and RGS10-null mouse spleens, as determined by flow cytometry. **b** Percentage of CD4+ T cells from WT or RGS10 null mice (*n* = 3) that migrated to the lower chamber containing serum free-medium (VEH), CXCL12 (10 nM) or under control conditions (CXCL12 in both lower and upper chambers) *, *p* < 0.05, two-way ANOVA

The resistance of RGS10-null mice to EAE could be explained by a defect in generation or maintenance of autoreactive T cell populations; however, given the reduced frequency of T cells in the spinal cords of RGS10-null mice with EAE (Fig. 5), it is also possible that RGS10-null CD4+ T cells have defects in their ability to traffic into the CNS. Thus, we next examined whether RGS10 regulates CD4+ T cell migration to chemokines in vitro. We found that, compared to WT cells, RGS10-null CD4+ T cells displayed attenuated migration in response to CXCL12 (a ligand for CXCR4) (Fig. 6b) and CCL19 (aligand for CCR7) (data not shown). Notably, CXCR4 and CCR7 protein were expressed at similar levels in WT vs. RGS10-null CD4+ T cells, suggesting that attenuated migration is not due to abnormal receptor expression (Fig. 6a and data not shown). Rather, RGS10 may regulate signaling pathways downstream of chemokine receptors on CD4+ T cells.

### Impaired Th-1-mediated neuroinflammation in RGS10-null mice

**Fig. 7:**
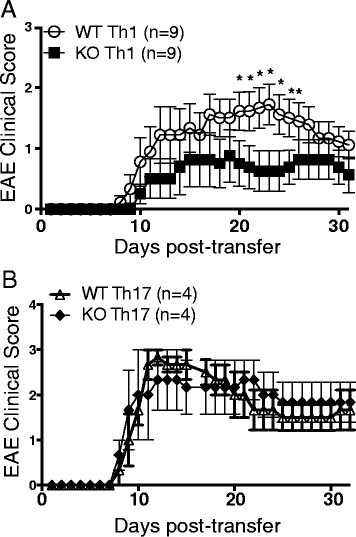
RGS10-null Th1 but not Th17 cells adoptively transferred into WT mice conferred attenuated EAE. RGS10-null or WT mice (*n* = 6) were immunized with MOG_35–55_/CFA._._ Nine days later, splenocytes and lymph node cells were harvested from RGS10-null and WT mice and then differentiated in vitro into Th1 cells or Th17 cells as described in the “Methods” section. Th1 or Th17 cells from RGS10-null or WT mice were injected into 5- to 6-week-old WT naïve recipient mice; mice were followed clinically up to at least day 32. Mean clinical scores (±SEM) of RGS10 WT adoptive transfer recipients of encephalitogenic Th1 (**a**) or Th17 (**b**) cells from MOG_35–55_-immunized WT and RGS10-null donors. *p < 0.05, Mann-Whitney *U* test

To determine whether the decreased EAE disease severity in the RGS10-null mice was due to autoreactive T cell-intrinsic defects, we performed adoptive transfer experiments. Encephalitogenic, MOG_35–55_-reactive Th1 or Th17 cells from WT or RGS10-null mice were transferred to WT recipient mice. Our results showed that the recipients of Th1 cells from RGS10-null EAE mice developed attenuated disease compared to mice that received WT Th1 cells. Conversely, the recipients of Th17 cells from RGS10-null mice developed clinical EAE that was statistically indistinguishable from recipients of WT Th17 cells. These findings suggest that a key determinant of the EAE phenotype in RGS10-null mice is impaired Th1 cell function and/or trafficking (Fig. 7).

## Discussion

Although EAE has limitations as a model for MS [36], studies of patient material and preclinical animal models support the notion that autoreactive T cells mediate the initial stages of MS pathology and that EAE models recapitulate key aspects of the myelin-reactive T cell response in MS [37–39]. Here, we utilized EAE not only as an MS model but also as a tool to elucidate roles for RGS10 in CD4+ T cell-mediated CNS autoimmune disease*.*

We previously showed that RGS10 plays a critical role in inflammatory microglial activation via negative regulation of NF-κB signaling [27]. Chronic peripheral administration of lipopolysaccharide (LPS) in RGS10-null mice caused chronic microgliosis and loss of dopaminergic (DA) neurons [26]. Therefore, our initial hypothesis was that RGS10-null mice would develop more severe EAE than their WT counterparts. Instead, we observed that RGS10-null mice displayed significantly milder EAE (Fig. 1 and Table 1). Moreover, there was less demyelination and leukocytic infiltration in CNS in RGS10-null EAE mice (Figs. 1 and 5). Lymphocytes from RGS10-null mice exhibited reduced proliferation and cytokine production in MOG_35–55_ recall assays (Fig. 4). Conversely, WT and RGS10-null DCs were comparable in their ability to stimulate proliferation of MOG_35–55_-reactive CD4+ T cells (Fig. 2). Our data suggest that RGS10 does not influence antigen presentation functions of DCs derived from peripheral lymphoid tissues, but we cannot exclude potential roles for RGS10 in governing APC functions within the CNS.

RGS10 has been reported to oppose the chemokine-stimulated signaling that is needed for T cell adhesion mediated by α4β1 and αLβ2 [40]. Thus, upregulation of adhesion to α4β1 and αLβ2 ligands in response to CXCL12 and CCL21 was significantly stronger in RGS10 deficient cells, suggesting that RGS10 inhibits integrin-mediated adhesion by repressing Gαi-dependent signaling [40]. CXCL12 (SDF-1α) is a pleiotropic chemokine that participates in the regulation of tissue homeostasis, immune surveillance, inflammatory responses, and cancer development (reviewed in [41]). Although our studies did not directly address the role of RGS10 in T cell migration in vivo, our data demonstrate that RGS10-null CD4+ T cells display attenuated migration to CXCL12 in in vitro transwell assays (Fig. 6). Thus, RGS10-null CD4+T cells and other RGS10-deficient immune cells might also display impaired migration during homing and/or infiltration into the CNS. Combined with defects in T cell activation and pathogenic Th cell differentiation within effector tissues (Figs. 4 and 5), a migration defect could account for the observed reduction in immune cell infiltration in the CNS of RGS10-null mice with EAE.

There are a number of immune-based therapeutic drugs available or in development for the treatment of MS. However, a major challenge for the field has been the inability to predict which treatment will work best for any given individual due to lack of mechanistic information about each individual’s disease [42]. Another pressing challenge in the field is that there is no therapy to specifically target pathogenic cells without disrupting the beneficial or disease-limiting components of the immune system. Indeed, many current MS drugs have broad effects on the immune system. For example, agents such as Tysabri (natalizumab, a monoclonal antibody (mAb) against the α4 integrin adhesion molecule) and Gilenya (fingolimod, a small molecule sphingosine-1-phosphate receptor modulator) significantly alter leukocyte homing, while Lemtrada (alemtuzumab, a mAb directed against CD52) depletes immune cells. These approaches can lead to significant side effects, which may include increased vulnerability to infections. Therefore, it is important to identify biomarkers that can better inform clinicians to choose the most appropriate treatment for a patient. A comprehensive understanding of which immune cell subsets are key contributors to pathogenicity and molecules that regulate their activity might lead to the development of novel and more effective treatments for MS.

The primary function of RGS proteins is believed to be the regulation of heterotrimeric G protein signaling at the plasma membrane. However, our findings as well as those of others [43–45] reveal that RGS proteins translocate to the nucleus and are found in high abundance at other intracellular sites. This suggests that RGS10 may have functions other than modulating G-protein signaling. In vitro-generated Th1 cells from RGS10 null mice induced EAE that, overall, was less severe than disease caused by WT T cells upon transfer into immunocompetent hosts, suggesting roles for RGS10 in regulating Th1-mediated autoimmune CNS inflammation (Fig. 7). However, we do not exclude T cell-independent roles for RGS10 in EAE. Indeed, RGS10 protein is expressed by various immune cells, including neutrophils, dendritic cells, and macrophages [34]. Future studies involving conditional deletion or enhancement of RGS10 in specific leukocyte subsets will provide additional opportunities to investigate this possibility in more depth.

## Conclusions

Our novel findings demonstrate a critical role for RGS10 in mediating autoimmune disease through regulation of lymphocyte function. This is the first study ever conducted to elucidate the function of RGS10 in effector lymphocytes in the context of EAE. The identification of RGS10 as an important regulator of inflammation will open possibilities for development of more specific targeted therapies for the treatment of MS and perhaps other chronic inflammatory neurological conditions.

## Abbreviations

CD: Crohn’s disease
EAE: experimental autoimmune encephalomyelitis
GAPs: GTPase accelerating proteins
GPCRs: G-protein-coupled receptors
LFB: Luxol fast blue
LN: lymph node
MOG: myelin oligodendrocyte glycoprotein
MS: multiple sclerosis
PBMCs: peripheral blood mononuclear cells
PMA: phorbol myristate acetate
RGS: regulator of G-protein signaling
UC: ulcerative colitis
